# Selective IgG2‐deficiency in yellow nail syndrome

**DOI:** 10.1002/rcr2.1102

**Published:** 2023-02-09

**Authors:** Steven Tessier, Santo Longo, Firas Ido

**Affiliations:** ^1^ Lewis Katz School of Medicine Temple University Philadelphia Pennsylvania USA; ^2^ Department of Internal Medicine St. Luke's University Health Network Bethlehem Pennsylvania USA; ^3^ Department of Pathology St. Luke's University Health Network Bethlehem Pennsylvania USA; ^4^ Department of Pulmonary and Critical Care St. Luke's University Health Network Bethlehem Pennsylvania USA

**Keywords:** bronchiectasis, empyema, immunoglobulin deficiency, pericardial effusion, yellow nail syndrome

## Abstract

IgG2‐deficiency increases susceptibility to recurrent pulmonary infections and the risk for bronchiectasis. Isolated IgG2‐deficiency has not been previously described in Yellow Nail Syndrome (YNS).

## Graphical abstract

We present a case of isolated IgG2‐deficiency in a patient with Yellow Nail Syndrome (YNS), manifested by yellow‐green nails, bilateral pleural effusions, pericardial effusion, bronchiectasis, and lower extremity edema. Given the rarity of the condition (estimated prevalence <1/1,000,000) and lack of thorough population‐based studies, association of new or rare manifestations with YNS relies heavily on individual case reports.

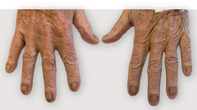

## CLINICAL IMAGE

**FIGURE 1:**
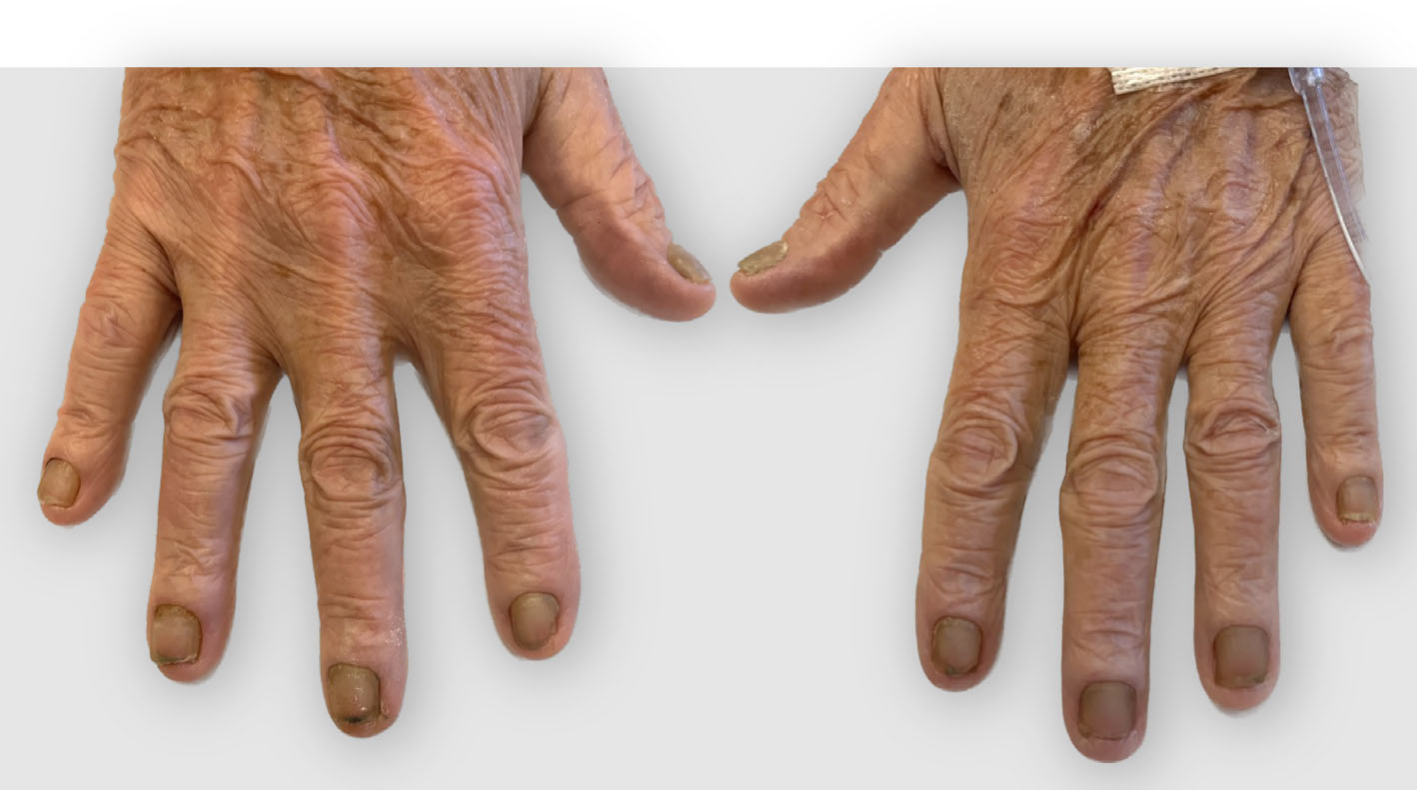
Image of the patient's hands. The nails are thickened and yellow‐green

**FIGURE 2:**
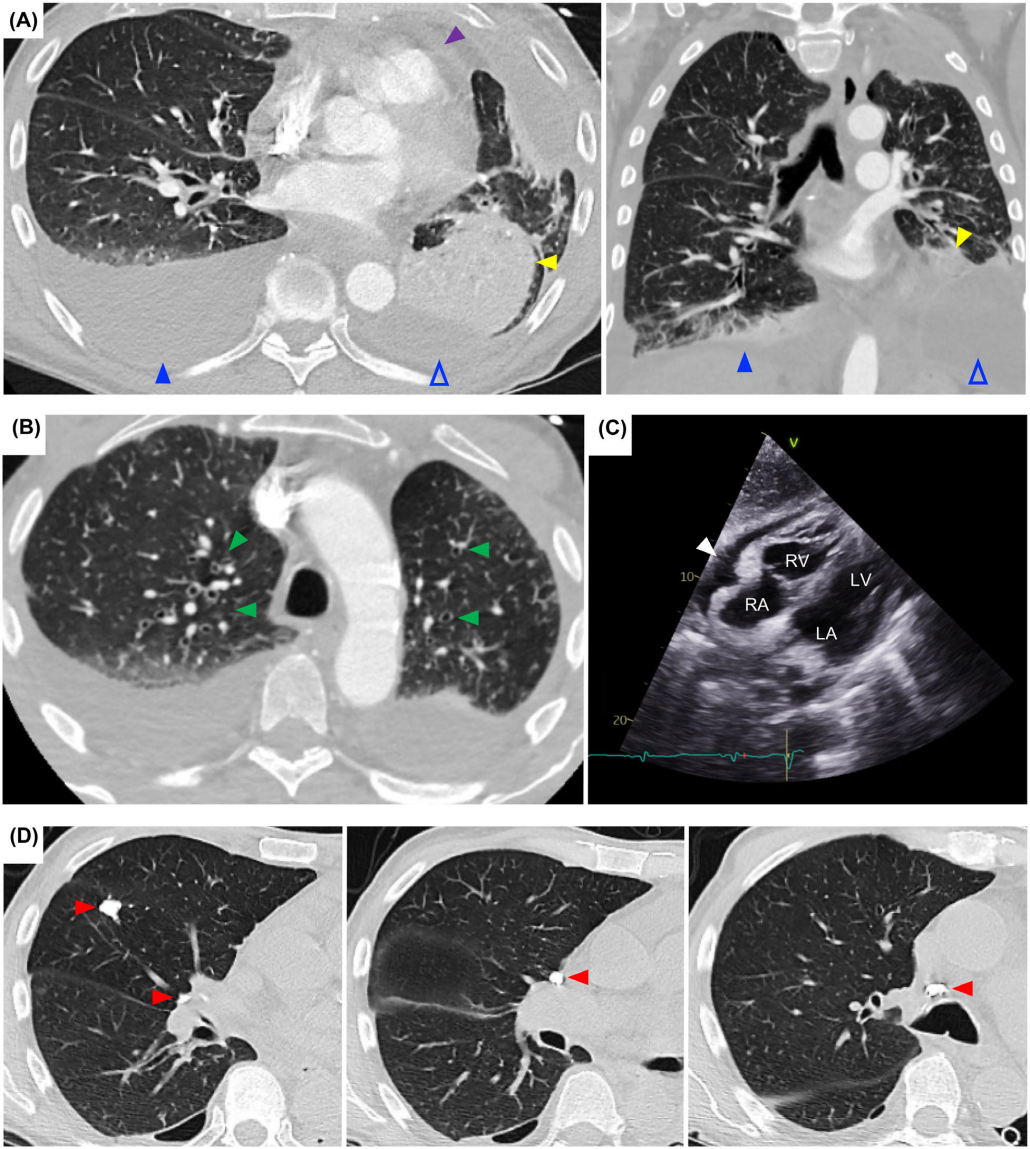
(A) Computed tomography angiography (CTA) showing moderate loculated left pleural effusion (open blue arrowheads), moderate free flowing right pleural effusion (filled blue arrowheads), rounded atelectasis in the left lower lobe (yellow arrowheads), and pericardial effusion (purple arrowhead). (B) CT without contrast showing bronchiectasis (green arrowheads). (C) Substernal view of transthoracic echocardiogram showing moderate pericardial effusion (white arrowhead) and right atrial wall inversion, concerning for tamponade. RA = right atria; RV = right ventricle; LA = left atria; LV = left ventricle (D) CT without contrast showing calcified granulomas (red arrowheads) in the right middle lobe and hilar/peritracheal lymph nodes

A 71‐year‐old man presented to the emergency department after experiencing a week of progressive shortness of breath and productive cough with yellow phlegm. His history included bilateral lower extremity non‐pitting edema refractory to diuretics, hospitalization for ileitis and cecitis associated with Giardia infection, mycoplasma pneumonia, chronic small pericardial effusion, and transudative pleural effusions that required multiple thoracenteses 2‐years‐prior. All the patient's nails had yellow‐green discoloration (xanthonychia) (Figure 1). The nail plates were abnormally thickened, opaque, and hard (scleronychia). Chest computed tomography showed moderate bilateral pleural effusions, rounded atelectasis in the left lower lobe (Figure 2A), and bronchiectasis (Figure 2B). Moderate pericardial effusion (Figure 2C) and calcified granulomas were also present (Figure 2D). Blood cultures, urine antigen, and pleural fluid from left‐side thoracentesis were positive for *Streptococcus pneumoniae*. Nail clipping specimens from the hands were negative for onychomycosis by periodic acid‐Schiff stain. In absence of other underlying etiologies, the patient was held to have Yellow Nail Syndrome (triad: yellow nails, respiratory disease, lymphedema).^1^ IgG and IgG2 levels drawn during an infection‐free period were 546 mg/dL (reference range: 603–1613 mg/dL) and 42 mg/dL (reference range: 130–555 mg/dL), respectively. YNS (estimated prevalence <1/1,000,000) has been rarely associated with immunodeficiency disorders, including IgG‐deficiency and common variable immunodeficiency, and successfully treated with IVIG.^1^, ^2^

## AUTHOR CONTRIBUTIONS

Conception and design: Firas Ido, Steven Tessier. Acquisition of data: Firas Ido, Steven Tessier. Analysis and interpretation of data: Firas Ido, Steven Tessier, Santo Longo. Drafting of manuscript: Firas Ido, Steven Tessier, Santo Longo. Revision of manuscript: Firas Ido, Steven Tessier, Santo Longo. Final approval of manuscript: Firas Ido, Steven Tessier, Santo Longo. All authors agree to be accountable for all aspects of the work

## CONFLICT OF INTEREST STATEMENT

None declared.

## ETHICS STATEMENT

The authors declare that appropriate written informed consent was obtained for the publication of this manuscript and accompanying images.

## Data Availability

The data that support the findings of this study are available from the corresponding author upon reasonable request.
